# Quantitative Analysis of Plant Cytosolic Calcium Signals in Response to Water Activated by Low-Power Non-Thermal Plasma

**DOI:** 10.3390/ijms231810752

**Published:** 2022-09-15

**Authors:** Enrico Cortese, Alessandro Galenda, Alessia Famengo, Luca Cappellin, Marco Roverso, Alessio G. Settimi, Manuele Dabalà, Diego De Stefani, Alessandro Fassina, Gianluigi Serianni, Vanni Antoni, Lorella Navazio

**Affiliations:** 1 Department of Biology, University of Padova, Via U. Bassi 58/B, 35131 Padova, Italy; 2 National Research Council, Institute of Condensed Matter Chemistry and Technologies for Energy (CNR-ICMATE), Corso Stati Uniti 4, 35127 Padova, Italy; 3 Department of Chemical Sciences, University of Padova, Via F. Marzolo 1, 35131 Padova, Italy; 4 Department of Industrial Engineering, University of Padova, Via F. Marzolo 9, 35131 Padova, Italy; 5 Department of Biomedical Sciences, University of Padova, Via U. Bassi 58/B, 35131 Padova, Italy; 6 Consorzio RFX, Corso Stati Uniti 4, 35127 Padova, Italy; 7 National Research Council, Institute for Plasma Science and Technology (CNR-ISTP), Corso Stati Uniti 4, 35127 Padova, Italy

**Keywords:** aequorin, *Arabidopsis thaliana*, chemical analyses, cytosolic Ca^2+^ transients, dielectric barrier discharge, plant calcium signalling, plasma activated water, plasma torch

## Abstract

Non-thermal plasma technology is increasingly being applied in the plant biology field. Despite the variety of beneficial effects of plasma-activated water (PAW) on plants, information about the mechanisms of PAW sensing by plants is still limited. In this study, in order to link PAW perception to the positive downstream responses of plants, transgenic *Arabidopsis thaliana* seedlings expressing the Ca^2+^-sensitive photoprotein aequorin in the cytosol were challenged with water activated by low-power non-thermal plasma generated by a dielectric barrier discharge (DBD) source. PAW sensing by plants resulted in the occurrence of cytosolic Ca^2+^ signals, whose kinetic parameters were found to strictly depend on the operational conditions of the plasma device and thus on the corresponding mixture of chemical species contained in the PAW. In particular, we highlighted the effect on the intracellular Ca^2+^ signals of low doses of DBD-PAW chemicals and also presented the effects of consecutive plant treatments. The results were discussed in terms of the possibility of using PAW-triggered Ca^2+^ signatures as benchmarks to accurately modulate the chemical composition of PAW in order to induce environmental stress resilience in plants, thus paving the way for further applications in agriculture.

## 1. Introduction

Calcium signalling constitutes a universal and versatile transduction mechanism across the tree of life, ranging from procaryotic to eukaryotic organisms [1,2,3,4,5,6]. In plants, it plays a fundamental role as a second messenger in a plethora of signalling pathways, including those activated by a wide variety of abiotic stresses (e.g., drought, salinity, cold/heat and oxidative stress [3,7,8,9]) as well as biotic stimuli, such as during pathogenic and symbiotic plant–microbe interactions [10,11,12,13]. 

We have recently demonstrated the induction of rapid and sustained cytosolic Ca^2+^ elevations in the model plant *Arabidopsis thaliana* (Arabidopsis) in response to plasma-activated water (PAW) generated by plasma torches, providing evidence for the involvement of Ca^2+^ signalling in the perception of PAW by plants [14]. In general, PAW can be produced by non-thermal atmospheric plasmas, often named cold plasmas, which are characterized by relatively weak ionization and by an electron temperature much higher than that of ions. This condition favours the presence of excited atoms and molecules which, when interacting with water, result in a rich mixture of short-, medium- and long-term chemicals, including reactive oxygen and nitrogen species. The mix and the concentration (thereafter called “the dose”) of the chemical species depends crucially on the plasma parameters and have been shown to stimulate biological responses when applied to living systems [15,16]. In agriculture, the resulting PAW has been recently emerging as a safe, rather inexpensive and eco-friendly technology that may reduce the use of pesticides and fertilizers, thanks to its antimicrobial/disinfection properties, as well as to the reported effects on the improvement of seed germination and plant growth [15,17]. Moreover, the ability of PAW to mildly induce plant defences, effectively boosting both plant resistance and resilience against subsequent harsh environmental conditions and pathogen attacks—a favourable pre-alert state termed “priming”—is leading research towards the fine-tuning of this novel “green” technology to maximise its beneficial effects in the context of more sustainable agriculture [18,19,20,21,22]. 

To analyse if Ca^2+^-mediating signalling could be regarded as a general mechanism of PAW sensing by plants and, in particular, to investigate the onset and the effect of mild treatments of plants with water activated by plasma discharges, in this work, we extended our previous analyses by using PAW generated via a different plasma device, i.e., an atmospheric dielectric barrier discharge (DBD). Indeed, a DBD plasma source can operate at low power and allows the plasma parameters to be finely tuned, thus enabling an accurate investigation into the effects of PAWs characterized by a moderate content of reactive chemical species. Aequorin-based Ca^2+^ measurement assays, conducted in transgenic Arabidopsis seedlings in response to DBD-PAW, confirmed the involvement of Ca^2+^ in the signal transduction mechanisms underlying plants’ beneficial responses to PAW. Quantitative analyses of the cytosolic Ca^2+^ signatures generated in plants by the administration of PAW produced via DBD, in comparison with that produced by using a plasma torch, unravelled the strict dependence of plants’ Ca^2+^-mediated responses on the different plasma sources, as well as on their specific operational conditions. Moreover, the subsequent treatment of plants with DBD-PAW highlighted a progressive strengthening of the Ca^2+^ response, possibly underlying a priming effect towards an increased plant stress resilience state. From this perspective, the collection of an extensive database concerning PAW-induced Ca^2+^ dynamics in plants will allow for a clear-cut understanding of how to finely modulate PAW generation, in order to effectively drive this innovative technology towards the desired outcomes on plant physiology. 

## 2. Results

### 2.1. Water Activated by Dielectric Barrier Discharge Induces Changes in the Cytosolic Concentration of Ca^2+^ in Arabidopsis thaliana

We have recently demonstrated the involvement of Ca^2+^ as an intracellular messenger in the plants’ perception of PAW generated by two different plasma torches [14]. In this work, we focused on the effect of PAWs characterized by relatively low doses of reactive chemical species, which could not be investigated with the previous experimental set-up, as the torches operate only at high power. 

**Figure 1:**
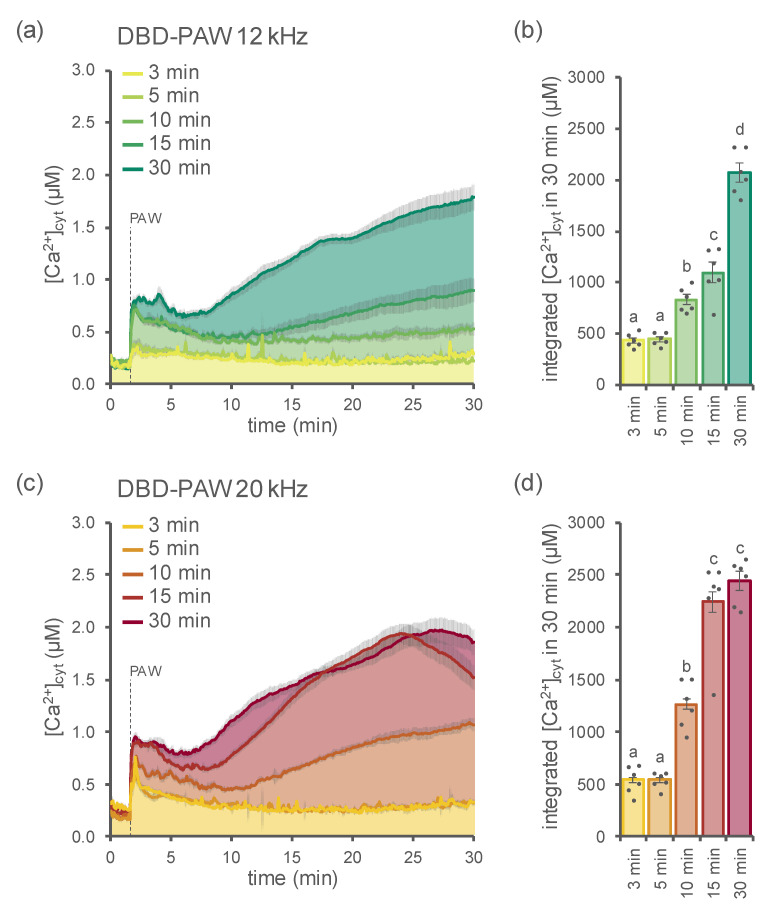
Monitoring of cytosolic Ca^2+^ signals triggered in *Arabidopsis thaliana* (Arabidopsis) in response to DBD-PAW. Variations in the cytosolic Ca^2+^ concentration ([Ca^2+^]_cyt_) were measured in 1-week-old Arabidopsis seedlings stably expressing aequorin in the cytosol after the administration (at 100 s, dashed line) of 1:2 dilutions of various DBD-PAWs. The DBD’s discharge frequency was set either at 12 kHz (green shades (**a**,**b**)) or 20 kHz (red shades (**c**,**d**)); for each setting, five different DBD-PAWs were obtained by exposing deionized H_2_O to DBD cold plasma for increasing time intervals (from 3 to 30 min). (**a**,**c**) Data are the means (solid lines) ± SE (shading) of the [Ca^2+^]_cyt_ dynamics induced in 6 independent seedlings. (**b**,**d**) Integrated [Ca^2+^]_cyt_ dynamics over 30 min. Each dot represents a single biological replicate. Statistical analyses were performed according to Student’s *t*-test (*p* < 0.05), with different letters indicating significant differences.

To monitor and precisely measure changes in cytosolic Ca^2+^ ([Ca^2+^]_cyt_), 1-week-old seedlings of *Arabidopsis thaliana* stably expressing the bioluminescent Ca^2+^ reporter aequorin in the cytosol [23,24,25] were challenged with PAWs generated by DBD (hereafter called DBD-PAW) under different operational conditions. In particular, two distinct plasma discharge frequencies (12 kHz (Figure 1a,b) and 20 kHz (Figure 1c,d)) were used, as well as five different time intervals of exposure of deionized H_2_O to DBD, ranging from 3 to 30 min (Figure 1a–d). 

In all cases, the DBD-PAWs were found to be able to induce cytosolic Ca^2+^ signals (Figure 1), whereas the application of deionized H_2_O (control, not exposed to cold plasma) did not trigger any detectable change in [Ca^2+^]_cyt_ (Figure S1a,b). The dynamics of the DBD-PAW-induced [Ca^2+^]_cyt_ elevations were shown to strictly depend on the duration of the plasma treatment (Figure 1a–d), with prolonged exposures (15 and 30 min) leading to sustained Ca^2+^ elevations, while short time intervals (3 and 5 min) were responsible for the induction of [Ca^2+^]_cyt_ transients that quickly dissipated within 10 min. The Ca^2+^ transients observed with the 10 min DBD-PAW showed an intermediate Ca^2+^ dynamics at both frequencies, thereby representing a transition state between the short and long treatments.

Moreover, the magnitude of the elicited [Ca^2+^]_cyt_ elevations (measured as the integration of the [Ca^2+^]_cyt_ values over 30 min) was found to correlate with the plasma discharge frequency settings of the DBD device, indicating the occurrence of significantly more intense Ca^2+^ signals in the samples treated with DBD-PAW at 20 kHz, in comparison with those treated with DBD-PAWs at 12 kHz, at least for the medium and long time intervals (10, 15 and 30 min) (Figure S2).

Measurements of the pH for each of the abovementioned DBD-PAWs revealed their moderate acidic nature for both of the discharge frequency conditions applied (Figure S3) as a result of the acidification of deionized H_2_O (Figure S1c), caused by exposure to cold plasma. The pH of the various DBD-PAWs was also found to strictly correlate with the time interval of the cold plasma treatment (Figure S3), in a fashion similar to the behaviour observed in terms of the triggered Ca^2+^ signals (Figure 1).

Furthermore, by comparing the DBD-PAWs obtained with the same time interval of exposure to plasma at different discharge frequencies (12 kHz and 20 kHz), the measured pH values indicated a more pronounced acidity for PAW samples generated at 20 kHz in comparison with those obtained at 12 kHz (Figure S4).

### 2.2. Determination of Nitrate and Nitrite Content in DBD-PAWs 

**Figure 2:**
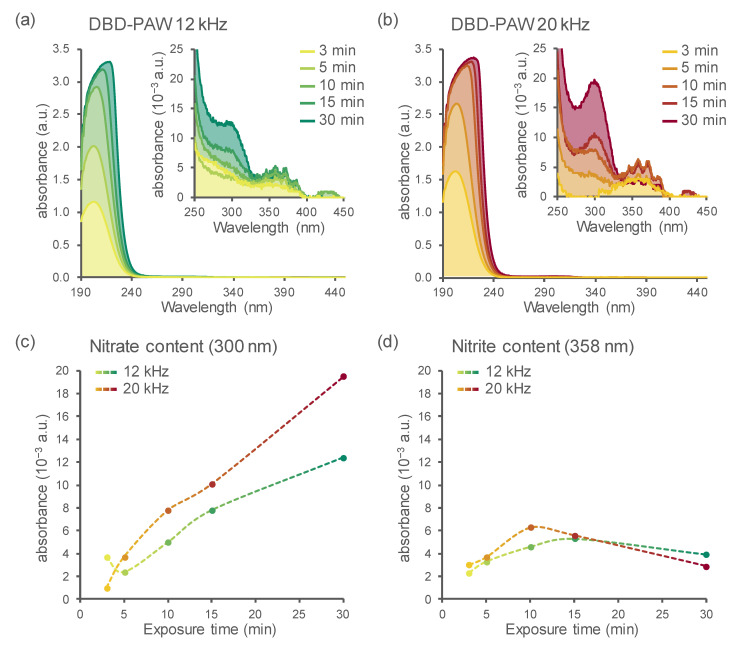
Detection of the main nitrogen species in DBD-PAW by spectrophotometric analyses. UV–Vis spectral analyses were performed on DBD-PAWs obtained at different discharge frequencies: (**a**) 12 kHz (green shades); (**b**) 20 kHz (red shades). DBD-PAWs were obtained by progressively increasing the exposure time of deionized H_2_O to DBD cold plasma (from 3 to 30 min). The inset magnifications (**a**,**b**) reveal the presence of distinctive bands in the regions related to nitrates (NO_3_^−^) and nitrites (NO_2_^−^). Data shown in (**a**,**b**) are representative traces (solid lines and areas under the curve) of the absorbance spectra in the 190–450 nm region, which were quantified at 300 nm (**c**) and 358 nm (**d**) to provide comparative estimations for NO_3_^−^ and NO_2_^−^ contents among the distinct DBD-PAWs.

Spectrophotometric analyses performed immediately after the generation of the various DBD-PAWs allowed for the detection of the amount of nitrogen-containing species: the absorbance spectra of all the samples revealed the presence of relative peaks centred on the regions related to nitrates (300 nm) and nitrites (358 nm) (Figure 2a,b and insets). Quantified values for each sample were then plotted according to the DBD’s operational parameters, showing a clear correspondence between the duration of plasma–water exposure and the NO_3_^−^ enrichment in DBD-PAW (Figure 2c). NO_2_^−^ content (mainly present as HNO_2_, as demonstrated by the structured band shape) instead seemed to increase in the first 10 or 15 min (for 20 and 12 kHz settings, respectively) of the cold plasma treatment and then decrease (even drastically, in the case of DBD-PAW generated at 20 kHz) when the plasma activation was protracted up to 30 min (Figure 2d). 

The presence of dissolved ozone in DBD-treated water cannot be excluded because of the well-known characteristic odour after the treatment and the masked shoulder at about 260 nm [26]. Nevertheless, the amount of ozone could not be quantified because of the superimposition of the nitrate signal (band at 300 nm) and the contributions of nitrite and nitrate ions at 190–240 nm.

**Figure 3:**
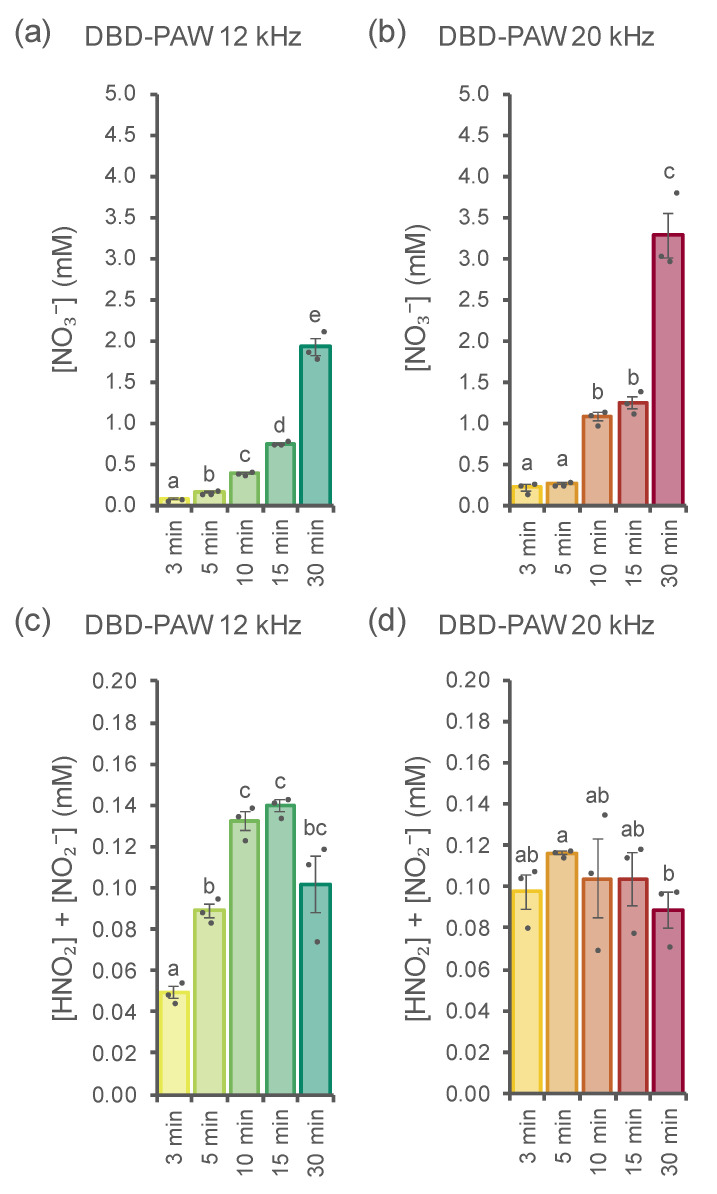
Quantification of the main nitrogen species in DBD-PAW. Ion chromatography analyses were performed on DBD-PAWs obtained at different discharge frequencies: (**a**,**c**) 12 kHz (green shades); (**b**,**d**) 20 kHz (red shades). DBD-PAWs were obtained by progressively increasing the exposure time of deionized H_2_O to the DBD cold plasma (from 3 to 30 min). Data are the means ± SE of 3 independent samples (dots). Statistical analyses were performed according to Student’s *t* test (*p* < 0.05), with different letters indicating significant differences.

Additional investigations were performed in order to isolate and measure the main nitrogen species generated by the cold plasma–water interaction: ion chromatography analyses of the DBD-PAWs allowed us to precisely quantify their contents of nitrates ([NO_3_^−^]) and nitrites (measured as the sum of [HNO_2_] and [NO_2_^−^]), showing a clear correspondence between PAW exposure time and nitrate abundance for DBD-PAWs obtained at plasma discharge frequencies of 12 kHz (each timepoint was statistically different from the others) and 20 kHz (the timepoints appeared to be grouped together, with statistical differences evident only when comparing samples across short (3 and 5 min), medium (10 and 15 min) and long (30 min) time intervals) (Figure 3a,b). Concerning nitrites, treatments at the two distinct frequency conditions led to different outcomes instead: DBD-PAWs generated at 12 kHz still showed a gradual increase in the nitrite concentration if we consider the first three time intervals, while no variance was found among samples exposed to cold plasma for 10, 15 and 30 min (Figure 3c); however, the nitrite concentration values for DBD-PAWs obtained at 20 kHz were all found to be similar, with the only statistical relevance resulting from the analyses of the 5 and 30 min treatments (Figure 3d).

Side-by-side comparisons of nitrogen species measurements in 12 and 20 kHz DBD-PAWs allowed us to observe that NO_3_^−^ concentrations at each fixed time always differed between the two discharge frequency settings (Figure S5a–e), while the same was observed for nitrite content only in the samples relative to the 3 min and 5 min cold plasma treatments (Figure S5f,g): the remaining time intervals considered were not found to be statistically different in terms of nitrite abundance (Figure S5h–j).

The data and data trends shown in Figure 2 and Figure 3 allow some speculation about the dynamics of the PAW’s composition and evolution with treatment type and time. The nitrite-related signals appeared to reach a relative maximum after 15 min of exposure at 12 kHz, while the same maximum was reached after 10 min when the DBD operated at 20 kHz, thus confirming the higher effectiveness of the treatment at 20 kHz. Nitrite content started to decrease for the subsequent exposure times. On the other hand, nitrate content showed a monotone non-linear increase. It is well known that nitrous acid is unstable in solution and shows dismutation, thus producing NO_x_ and HNO_3_. This can help justify the more than linear increase in nitrate content for the longer treatments. 

### 2.3. Subsequent DBD-PAW Treatments Triggered Slower and Stronger Plant [Ca^2+^]_cyt_ Elevations

In response to several elicitor treatments, plants can respond by inducing a temporary refractory state, i.e., the failure to react to a second dose of the same stimulus [27,28,29,30]. To investigate the Ca^2+^ response of Arabidopsis seedlings to consecutive treatments with DBD-PAW, plants were challenged with a second equivalent dose of freshly generated DBD-PAW (H_2_O exposed to DBD cold plasma for 3 min at 20 kHz), at different time intervals (15, 60 and 120 min) after the first treatment. 

For the present analysis, PAW obtained by exposing deionized H_2_O to cold plasma for a short time interval (3 min) was selected (among all the numerous conditions considered in this work) in order to avoid triggering sustained [Ca^2+^]_cyt_ dynamics that could mask the effect of the second PAW administration.

**Figure 4:**
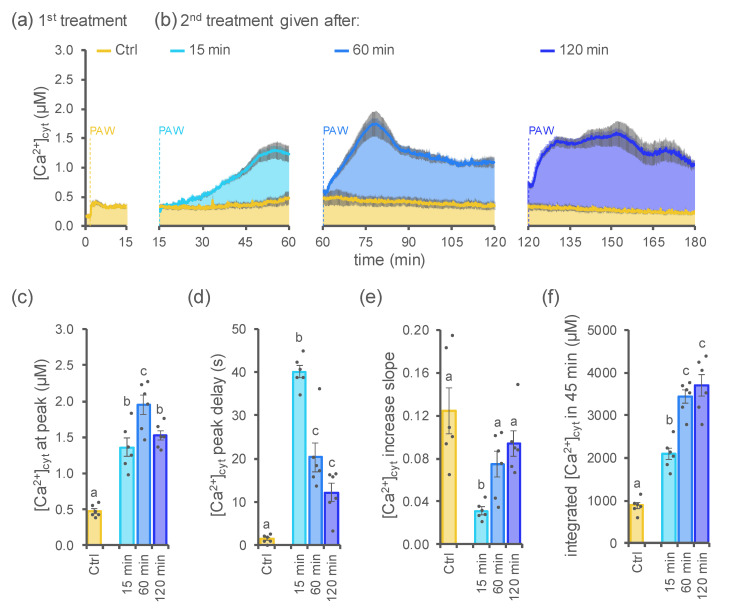
Subsequent administration of DBD-PAW to Arabidopsis seedlings triggered a modified Ca^2+^ signature, depending on the resting time between the two treatments. Elevations in [Ca^2+^]_cyt_ were measured in aequorin-expressing Arabidopsis seedlings after the administration (at 100 s, yellow dashed line) of a 1:2 dilution of DBD-PAW (**a**). A second equivalent dose of DBD-PAW was administered (dashed lines) at different times (**b**): 15 min (light blue), 60 min (blue) and 120 min (dark blue). All DBD-PAWs were obtained by exposing deionized H_2_O to cold plasma for 3 min, with the DBD’s discharge frequency set at 20 kHz. (**a**,**b**) Data are the means (solid lines) ± SE (shadings) of the evoked [Ca^2+^]_cyt_ dynamics in 6 independent seedlings. Control seedlings (Ctrl) were subjected to only one DBD-PAW treatment. (**c**–**f**) Statistical analyses of the [Ca^2+^]_cyt_ peak (**c**), the temporal delay in the onset of the [Ca^2+^]_cyt_ peak (**d**), the slope of the [Ca^2+^]_cyt_ increase (**e**) and the integrated [Ca^2+^]_cyt_ dynamics over 45 min (**f**). Each dot represents a single biological replicate. Bars labelled with different letters differ significantly (Student’s *t*-test, *p* < 0.05).

Measurements of [Ca^2+^]_cyt_ variations revealed that the second treatment resulted in the induction of Ca^2+^ signals characterized by an increased magnitude, as well as a slower onset (Figure 4). Indeed, the second dose-triggered [Ca^2+^]_cyt_ peak was found to be significantly higher (Figure 4b,c,f) compared with that evoked by the first DBD-PAW application (Figure 4a). Moreover, the triggering of Ca^2+^ signals after the second DBD-PAW treatment was shown to be delayed (Figure 4b,d,e) with respect to the rapidly induced [Ca^2+^]_cyt_ peak in response to the first DBD-PAW administration; however, it is noteworthy that the mean delay for the onset of the second dose-induced [Ca^2+^]_cyt_ peak appeared to be progressively lower for samples with protracted resting times (15, 60 or 120 min) between the two DBD-PAW applications, which can also be inferred from the progressively increased slope of the [Ca^2+^] increase trace (Figure 4d,e). Integrated Ca^2+^ signals after the administration of the second DBD-PAW dose were all characterized by a significant increase in their magnitude in contrast to the low-intensity Ca^2+^ signal evoked by the first DBD-PAW application: in particular, seedlings treated with the second DBD-PAW dose after either 60 or 120 min resulted in the maximum [Ca^2+^]_cyt_ increase among the tested conditions (Figure 4f). Taken together, these data suggest that plants retain a cellular “memory” of the first treatment with PAW, which is attested by the change in the Ca^2+^ signature triggered by subsequent administration of the same stimulus. These data indicate that modified Ca^2+^ signalling may underlie the progressive setting-up of plant adaptation mechanisms towards more efficient adaptive responses to environmental stresses. 

### 2.4. Comparison of the [Ca^2+^]_cyt_ Signatures Induced in Plants by PAW Generated by Either a DBD or a Plasma Torch

**Figure 5:**
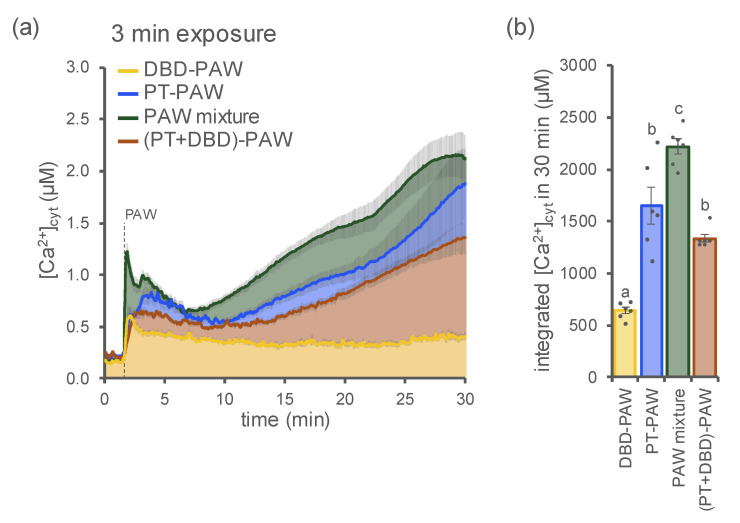
Comparison of the Ca^2+^ signatures induced by PAWs generated by different cold plasma sources. [Ca^2+^]_cyt_ elevations were measured in aequorin-expressing Arabidopsis seedlings after the administration (at 100 s, dashed line) of PAW generated by a DBD (DBD-PAW, yellow, 1:4 dilution), PAW generated by a plasma torch (PT-PAW, blue, 1:4 dilution), a PAW mixture composed of equal volumes (1:1) of both DBD-PAW and PT-PAW (green, 1:2 dilution), a double-treated PAW obtained by sequentially exposing deionized H_2_O to PT and DBD cold plasmas ((PT+DBD)-PAW, brown, 1:4 dilution). All PAWs were obtained by exposing deionized H_2_O to cold plasma for 3 min, with the DBD’s discharge frequency set at 20 kHz. (**a**) Data are the means (solid lines) ± SE (shadings) of the evoked [Ca^2+^]_cyt_ dynamics in 6 independent seedlings. (**b**) Integrated [Ca^2+^]_cyt_ dynamics over 30 min. Each dot represents a single biological replicate. Statistical analyses were performed according to Student’s *t*-test (*p* < 0.05), with different letters indicating significant differences.

In order to compare the plants’ Ca^2+^ responses to PAW generated by different plasma sources, we monitored the [Ca^2+^]_cyt_ in Arabidopsis seedlings treated with samples obtained by exposing H_2_O to either a DBD (DBD-PAW, 20 kHz) or a plasma torch (PT-PAW, 900 W) for the same time interval (3 min). Striking differences were found both in terms of the dynamics and amplitude of the evoked [Ca^2+^]_cyt_ signals. In particular, in aequorin-expressing Arabidopsis plants, PT-PAW triggered a much more pronounced and sustained cytosolic Ca^2+^ elevation [14] compared with the Ca^2+^ signature induced in the same experimental system by DBD-PAW (Figure 5).

When PAWs were generated by the abovementioned plasma-generating devices and mixed together in equal volumes upon production, then administered to Arabidopsis seedlings as a PAW mixture, the resulting elevation in [Ca^2+^]_cyt_ was characterized by an increased amplitude, in comparison with those evoked by the single DBD-PAW and PT-PAW (Figure 5). These data support the notion that the plant Ca^2+^ response is strictly dependent on the unique chemical content of the PAW, which, in turn, correlates with the cold plasma source used for PAW generation.

When Arabidopsis seedlings were treated with a PAW sample obtained by sequentially exposing H_2_O for 3 min to PT cold plasma and then to DBD cold plasma, it was found that the double activation with the two distinct plasma devices did not induce the same Ca^2+^ signature as the one triggered in response to the PAW mixture, showing instead an elevation in Ca^2+^ of significantly reduced magnitude (Figure 5). Indeed, the plant [Ca^2+^]_cyt_ increase in response to the application of the twice-exposed sample was very similar to the Ca^2+^ trace induced by PT-PAW alone (Figure 5). This result suggests that the second activation of PAW—performed with the DBD device—was not able to significantly affect the already enriched chemical environment resulting from the first activation of H_2_O achieved with the plasma torch.

**Figure 6:**
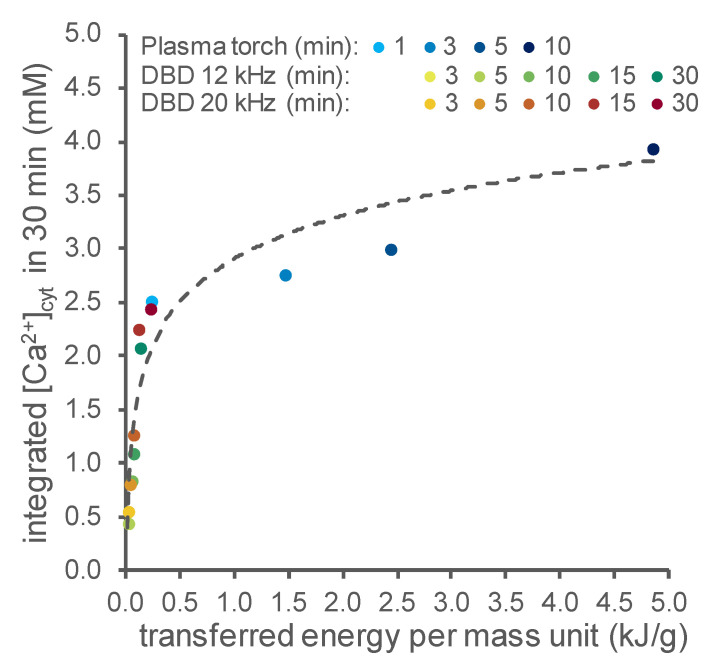
PAW-triggered cytosolic Ca^2+^ signals in Arabidopsis versus the total energy transferred to PAW. [Ca^2+^]_cyt_ dynamics were measured in aequorin-expressing Arabidopsis seedlings after the administration of 1:2 dilutions of either PT-PAW (blue shades) or DBD-PAW. The DBD’s discharge frequency was set either at 12 kHz (green shades) or 20 kHz (red shades). For each set-up, various PAWs were obtained by exposing deionized H_2_O to cold plasma for increasing time intervals (PT-PAW: from 1 to 10 min; DBD-PAW: from 3 to 30 min). Data are the means of the integrated [Ca^2+^]_cyt_ signals over 30 min in 6 independent seedlings and are plotted against the total energy per unit of mass transferred to water during the various cold plasma treatments.

The data showed that at longer activation times, DBD-PAW induced Ca^2+^ responses similar to those triggered by PT-PAW, despite the difference in the power of the distinct sources (the plasma torch operates at a nominal power of 900 W, whereas the DBD’s nominal power is in the range of 10–100 W). To understand the reason behind this experimental evidence, as a common reference parameter, we chose the energy transferred to the water, which takes both the power and activation time into account. Indeed, the transferred energy can be considered in the first approximation as a proxy of the chemical activation of the water and therefore of the chemical content. This parameter can be obtained using the proper heat transport equation, as reported below in the Materials and Methods section, if the water temperature is known. Therefore, the temperature of the water was measured before and immediately after exposure to the plasma, thus allowing the effective power and therefore the transferred energy to be evaluated by multiplying it by the activation time. Figure 6 shows the magnitude of the cytosolic Ca^2+^ signals evoked in Arabidopsis versus the transferred energy per unit of mass after activation by both cold plasma sources (DBD and PT). The results illustrate how sensitive the PAW-induced Ca^2+^ signalling is at lower energies—and therefore at lower doses of reactive chemical species in the PAW—whereas at higher doses, the magnitude of the induced Ca^2+^ increases tends to become saturated.

## 3. Discussion

Activation of water by atmospheric non-thermal plasma generates new chemical species, altering the water redox potential and changing its conductivity. The resulting plasma-activated water (PAW) is rich in reactive chemical species and is increasingly regarded as a promising new tool for a wide range of different applications, spanning from medical and dentistry treatments [16], to food preservation, as well as to the enhancement of agricultural practices [21,31]. The beneficial effects of plasma-activated water administration have been reported in numerous areas of plant science, with evidence showing increased seed germination rates and the promotion of plant growth, as well as antibacterial/antifungal activity, along with the recently reported improvement in plant resistance to pathogens (see [15,16,17,18,19,20,21] for reviews).

PAW administration to plants may therefore represent an attractive eco-sustainable alternative to reduce the excessive use of pesticides and chemical fertilizers in agriculture, therefore contributing to achieving the goals of the “Farm to Fork” strategy, aiming to cut the use of chemical pesticides by 50% at least by 2030 and to reduce the use of synthetic fertilizers by 20%. 

To elucidate the biochemical and molecular basis of the so-called plant “plasma vaccination” [18], we focused on investigating the link between PAW perception and the positive downstream responses of plants. In a recent study, we demonstrated that the treatment of Arabidopsis seedlings with water activated by a plasma torch induced rapid and sustained intracellular Ca^2+^ elevations [14]. In this work, we carried out a thorough analysis of the effects of water activated by a dielectric barrier discharge (DBD), at the level of the induction of changes in plant [Ca^2+^]_cyt_. In particular, a DBD was developed to perform operations at relatively low power, allowing investigations into the potential Ca^2+^-mediated perception of PAW generated by low-energy water activation. Indeed, the comparison between the cytosolic Ca^2+^ signals induced by PAW obtained by either the DBD or a plasma torch (PT) indicated that the Ca^2+^-inducing activity exerted by the PT was comparable with that of the DBD for longer activation times (and thus the transferred energy) and tended to become saturated, whereas for shorter activation times (and thus lower transferred energy) the behaviour was quite sensitive, even to small changes in this parameter.

In general, the DBD system is a versatile plasma source which, in principle, allows the power to be finely adjusted, so that a wide range of chemical mixtures at relatively low doses can be obtained and finely tuned. Moreover, it offers the advantage of being easily scalable, as the electrode surface can be tailored to the specific scope. The DBD was caged in a support structure for easy handling, so that it could operate close to the biological set up and/or the chemistry facilities, to minimize the time between the generation, plant treatment and chemical analysis steps. 

As an experimental system, we used a transgenic Arabidopsis line stably expressing the bioluminescent Ca^2+^ indicator aequorin. Aequorin-based Ca^2+^ measurements provide a sensitive and reliable assay to quantitatively monitor and analyse PAW-induced intracellular Ca^2+^ changes. The relative insensitivity of aequorin to pH [32] renders this genetically encoded Ca^2+^ indicator suitable for the accurate monitoring of Ca^2+^ variations in response to PAWs generated by different cold plasma sources, which are characterized by low pH and are incompatible with the proper functioning of most GFP-based Ca^2+^ probes [33]. The possibility that pH per se could be responsible for the observed Ca^2+^ changes in response to PAW has been previously ruled out [14].

Arabidopsis seedlings were treated with PAW generated by the DBD, using different frequencies of plasma discharge (12 and 20 kHz), as well as several exposure time intervals for the activation of the water. Ca^2+^ measurement assays demonstrated the induction of specific Ca^2+^ signatures, whose kinetic parameters were found to depend on the type of PAW. Notably, exposing water to DBD plasma discharge for 3–5 min resulted in the induction of transient [Ca^2+^]_cyt_ elevations that quickly dissipated, whereas longer time intervals of water exposure to DBD plasma (10, 15 and 30 min) induced higher and more prolonged Ca^2+^ increases. These results seem to indicate that the two different Ca^2+^ signature trends observed may underlie the different outcomes in the plant, suggesting a dose–response relationship to stressors according to the hormesis theory [34]. In particular, the low doses of reactive chemicals contained in the PAWs with a short time interval may be associated with stimulatory effects on plants, whereas the relatively higher doses of reactive species contained in the PAWs with a long time interval may be related to the process of priming, resulting in the pre-activation of plant defence responses. Increasing evidence suggests that the adaptive mechanisms of plants may rely on environmental hormesis, with specific effects dictated by mild and strong stressors, which can play fundamental roles in plant pre-conditioning and stress resilience [35]. From this perspective, plasma technology is emerging as a mechanism that is able to alleviate the adverse effects of both biotic and abiotic environmental stressors on plants [20]. Future pharmacological and genetic approaches will be directed at establishing a precise link between Ca^2+^-mediated signalling and specific downstream biological responses activated in plants by PAW.

The possibility of modulating the Ca^2+^ signals generated in plants upon treatment with PAW by simply controlling the operational setting of the plasma-generating device opens up intriguing possibilities about fine-tuning of the downstream responses of plants in terms of environmental stress resilience and/or the promotion of plant growth. The quantitative analysis of the intracellular Ca^2+^ signals triggered by the administration of PAW may allow us to establish a correlation between Ca^2+^ signatures and the observed beneficial effects on plants in the future. The collection of an extensive database of PAW-induced Ca^2+^ dynamics will pave the way for the establishment of a still lacking reference benchmark for the intrinsic features of PAW underlying their specific action on plant physiology. 

The induction of different Ca^2+^ signals by the range of tested DBD-PAWs is likely attributable to a complex mixture of different reactive chemical species contained in the PAWs. Future Ca^2+^ measurement experiments will be carried out in Arabidopsis seedlings in response to different mixtures of chemicals, in order to closely mimic and reproduce PAW-induced Ca^2+^ signatures. These Ca^2+^ assays will potentially identify new signalling molecules, ions or radicals able to activate Ca^2+^ signalling in plants.

Experiments performed by challenging plants with equal doses of DBD-PAW in temporal succession resulted in the induction of modified Ca^2+^ signatures. The obtained data suggest that plants, after the first treatment with PAW, respond to subsequent challenges with the same type of stimulus with a slightly delayed but significantly increased Ca^2+^ response. The results are coherent with desensitization and resensitization processes in Ca^2+^ signalling events, which have been reported for plant responses to both abiotic and biotic stresses [27,29,30]. It is known that plants retain a cellular “memory” of stress events, which is a mechanism allowing plants to better cope with subsequent similar events. Regulatory roles for Ca^2+^ signalling underlying plant memory setting and stress acclimation have recently been suggested [30,36]. 

The precise modulation of the physico-chemical configuration of PAW towards the desired properties may allow us to further innovate this cutting-edge technology by adding a whole new degree of customization, ultimately leading to cold plasma tailoring to specific agricultural needs, with great potential advantages for answering the contemporary challenges posed by climate change.

## 4. Materials and Methods

### 4.1. Generation of PAW by Using Different Plasma Sources: An Atmospheric Dielectric Barrier Discharge (DBD) and a Plasma Torch (PT)

**Figure 7:**
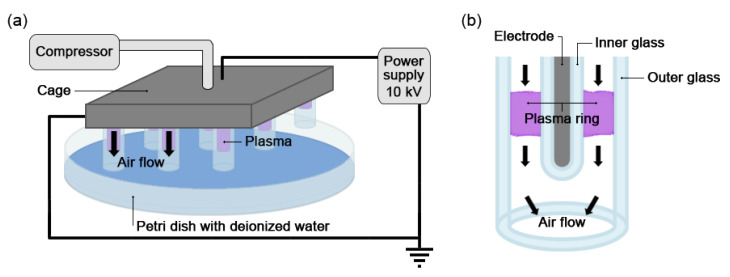
(**a**) Scheme of the dielectric barrier discharge (DBD) device and of the experimental set-up. (**b**) Details of one of the 13 electrode–glass tube components.

To carry out the investigations, a plasma source of the dielectric barrier discharge (DBD) type that operates at low power was developed. The DBD source consists of 13 copper electrodes, each covered by a closed glass tube (the inner glass tube) centred inside another glass tube (the outer glass tube) so that a ring of plasma is generated between the two glass tubes. A compressor provides a constant air flow through the subtle rings of cold plasma generated inside the tubes. The 13 glass tubes are equally distributed over a square area with 5 cm sided and caged in a support structure which ensures the stability of the set-up and allows the easy handling of the equipment. A schematic representation of the experimental set-up is shown in Figure 7a,b. A commercial pulsed power supply has been developed by AlmaSens (the high-tech electronic division of MONTRADE S.p.A., Bologna, Italy) and can generate voltage pulses from 2 to 20 kV, with a time width spanning from 300 to 1000 ns and a repetition rate from 2 to 20 kHz. In the present case, the power supply was set to 10 kV, a 800 ns pulse width and a frequency of either 12 or 20 kHz.

PAW was generated by exposing 20 mL aliquots of deionized water contained in a Petri dish to the plasma source. The source was placed 1 cm over the Petri dish so that the air activated by the cold plasma could interact with the water, generating the chemicals characteristic of PAW. The air flow was set in order to produce a gentle stirring of the water, which, combined with the chemical diffusion and a gentle resuspension of the liquid at the end of the activation process, allowed a uniform distribution of the chemicals in the PAW. The uniformity of the PAW was verified by measuring the pH in different samples of the same PAW. The experimental set-up was operated for various activation times (from 3 to 30 min) under a fume hood (speed: 0.35 m/s; flux: 856 m^3^/h), in order to avoid the effect of water vapour and to ensure standard and repeatable conditions of air flow.

To compare the present results with previous results at higher doses of PAW chemicals, the same plasma torch (PT#1) described in [14] was used. The torch was operated at 900 W for time intervals varying from 1 to 10 min and at an air pressure of 3 bar. In this case, the beaker with the deionized H_2_O sample (50 mL) was placed in an ice cooling bath to control the temperature increase during activation, and its surface was kept at 1.5 cm from the torch nozzle. The plasma plume generated was partially immersed in water and ensured a thorough mixing of the sample during the whole treatment.

### 4.2. DBD-PAW Chemical Analyses

It is known that the type and concentration of chemical species in PAW depend on the gases and liquids used to generate the plasma [17,37]. Therefore, accurate measurements of these quantities are fundamental for understanding the biological processes.

Spectrophotometric analyses were performed on freshly generated DBD-PAWs by using a double-beam UV–Vis spectrophotometer UV-2600 (Shimadzu, Kyoto, Japan). The instrument’s scan speed was set at 480 nm/min and quartz cuvettes with an optical path of 10 mm were used. Absorbance spectra for all the samples were collected in the range between 190 and 450 nm, each with a spectral resolution of 0.2 nm. 

Ion chromatography was used to quantify the nitrite and nitrate concentrations in the PAWs, using a Dionex ICS-6000 SP (Thermo Fisher Scientific, Waltham, MA, USA) with a Dionex IonPac ASIP-4 μm column (2 × 250 mm). The pH measurements were performed with BasiC 20 pH meter (Crison, Alella, Spain). 

### 4.3. Plant Material and Growth Conditions

As an experimental system we used a transgenic line of the model plant *Arabidopsis thaliana* (ecotype Columbia 0, Col-0) stably transformed with a construct encoding the chimera resulting from the fusion of the bioluminescent calcium indicator aequorin with the yellow fluorescent protein (Cyt-YA) [23]. Seeds derived from the F_3_ and successive generations were surface-sterilized and sown as previously described [38]. Sown plates were kept for 7 days at 21 °C at a 16 h–8 h light/dark cycle, after which, the aequorin-expressing seedlings were reconstituted overnight with 5 μM coelenterazine (Prolume, Pinetop, AZ, USA).

### 4.4. Monitoring of the Plant [Ca^2+^]_cyt_ Dynamics

Ten minutes prior to the [Ca^2+^]_cyt_ measurement assays, reconstituted seedlings were gently rinsed with deionized H_2_O to eliminate any residual excess coelenterazine, then placed individually in the wells of a CulturePlate-24 (PerkinElmer, Waltham, MA, USA) and allowed to rest floating in 150 μL of deionized H_2_O for an additional 10 min. PAW samples (or deionized H_2_O as a negative control) were applied to each seedling through injection with an EnVision 2105 XCite multimode plate reader (Perkin Elmer), which was also used to collect and integrate (over 500 ms) the emitted luminescence. The experiments were terminated by administering 150 μL of a discharge solution (1 M CaCl_2_, 30% (*v/v*) ethanol) to each well in order to fully consume the aequorin pool and properly calibrate the recorded light signals into [Ca^2+^]_cyt_ values, according to an algorithm based on the calibration curve of aequorin [39]. A high-throughput protocol was set up to perform the Ca^2+^ measurement assays in parallel, with a total of 6 seedlings processed at the same time: the luminescence deriving from each well was separately collected every 5 s, and the protocol was internally validated by comparing the calibrated [Ca^2+^]_cyt_ traces obtained in this way with those measured with a custom-built luminometer (ET Enterprises Ltd., Uxbridge, UK) [14].

### 4.5. Thermal Characterization of Cold Plasma Sources

The simplest method of estimating the energy transferred to the water is to consider the equation of heat transfer, taking the heating and loss terms into account. In particular, during the heating phase, the loss terms responsible for deviations from a linear increase were estimated from the time-resolved measurements of the water temperature. In this way, it is possible to use a unique parameter to compare the results obtained using the different plasma sources and the different experimental conditions, including the different power and activation times. Therefore, the following heat transport equation was applied:

(1) $$\frac{d\left(m\left(t\right)c_{s}T\left(t\right)\right)}{dt}=P_{in}-R_{cond}\left(T\left(t\right)-T_{env}\right)$$

where m(t) is the mass of the water (for the torch, this depends on the time, as some water was ejected from the beaker), $c_{s}$ is the specific heat of the water, T(t) is the temperature as a function of time; $P_{in}$ is the power applied by the plasma source to the water, $R_{cond}$ is the thermal resistance from the water to the environment and $T_{env}$ is the environmental temperature.

This equation has the following solution:

(2) $$T\left(t\right)=T_{0}e^{-Bt}+\frac{A}{B}\left(1-e^{-Bt}\right)\;\;;\;\;A=\frac{P_{in}+R_{cond}T_{env}}{m_{0}c_{s}}\;\;;\;\;B=\frac{R_{cond}}{m_{0}c_{s}}$$

where $m_{0}$ is the initial mass of the water (which was constant for the DBD).

If we compare the results for the different sources at different operational parameters and measure the temperature increase during the heating phase and the temperature decrease during the following decay, the effective power transferred to the water can be derived and, therefore, by multiplying by the activation time (i.e., the time for which the source was operating), the corresponding energy transferred to the water was obtained. 

## 5. Conclusions

The data obtained in this work demonstrate the Ca^2+^-mediated perception of PAW generated by a DBD in Arabidopsis seedlings. The quantitative analysis of the evoked Ca^2+^ dynamics highlighted significant correlations between the elicited Ca^2+^ responses and the DBD’s discharge frequency, activation time, as well as temporal delay between subsequent plant treatments. A comparison with the Ca^2+^ changes recorded after plant treatment with a different plasma-generating source, i.e., a plasma torch, was also presented and discussed. Our results, by providing fundamental insights into the sensing mechanisms of PAW by plants, may lay the ground for a better application of PAW in agriculture.

## Data Availability

The data presented in this study are available on request from the corresponding author.

## Supplementary Materials

The following supporting information can be downloaded at: https://www.mdpi.com/article/10.3390/ijms231810752/s1.
