# Supplementary material for: Quantitative Analysis of Plant Cytosolic Calcium Signals in Response to Water Activated by Low-Power Non-Thermal Plasma
Source: Int J Mol Sci. 2022 Sep 15;23(18):10752. doi: 10.3390/ijms231810752 (PMC9506352; doi:10.3390/ijms231810752)
Supplement: Supplementary file 1 [file ijms-23-10752-s001.zip › ijms-1865899-supplementary.pdf]

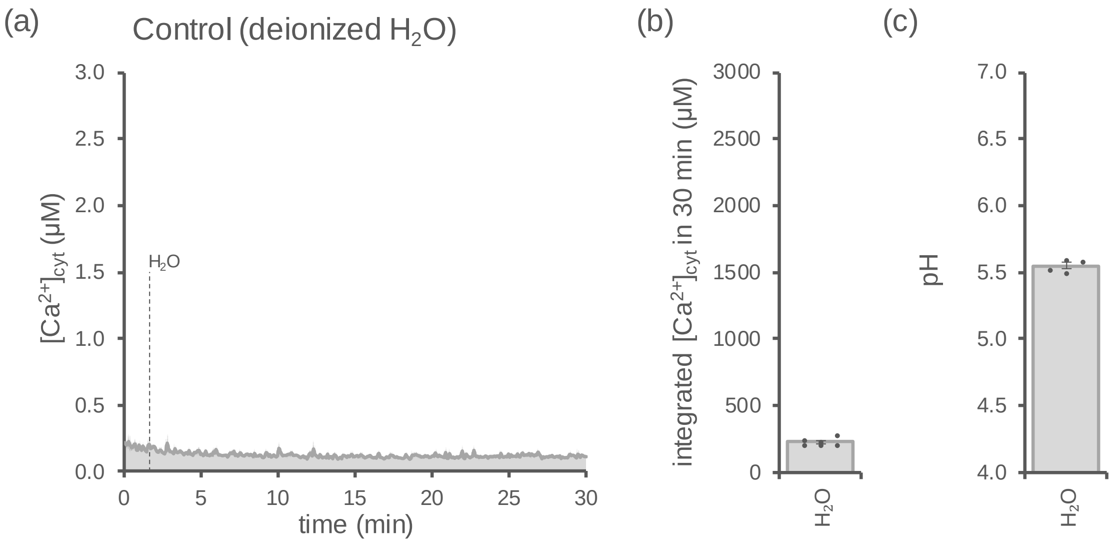

Figure S1. Monitoring of $[Ca^{2+}]_{cyt}$ in Arabidopsis seedlings in response to deionized H_2O. (**a**) No $[Ca^{2+}]_{cyt}$ variations were measured in aequorin-expressing Arabidopsis seedlings after the administration (at 100 s, dashed line) of an equal volume of deionized H_2O (control). (**b**) Integrated $[Ca^{2+}]_{cyt}$ dynamics over 30 min. (**a**,**b**) Data are the means (solid lines) ± SE (shading) of the $[Ca^{2+}]_{cyt}$ traces obtained in 6 independent seedlings. (**c**) Measurement of pH of deionized H_2O. Data are the means ± SE of 4 independent samples.

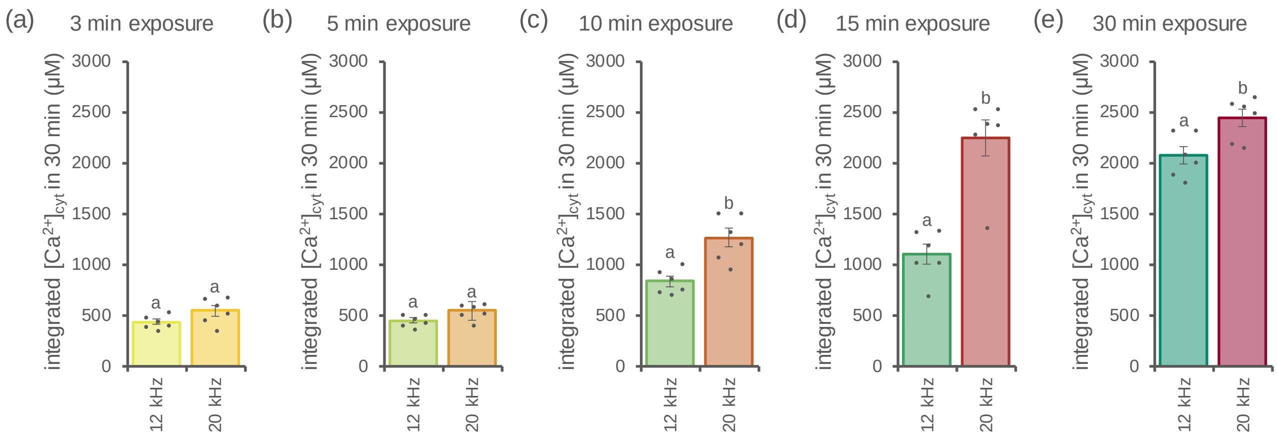

Figure S2. Comparison of the Ca^{2+} signal intensity triggered in Arabidopsis seedlings in response to DBD-PAWs generated at two distinct plasma discharge frequencies. $[Ca^{2+}]_{cyt}$ elevations were measured in aequorin-expressing Arabidopsis seedlings after the administration of 1:2 dilutions of various DBD-PAWs. DBD discharge frequency was set either at 12 kHz (green shades) or 20 kHz (red shades); for each setting five different DBD-PAWs were obtained by exposing deionized H_2O to DBD-cold plasma for increasing time intervals: 3 min (**a**), 5 min (**b**), 10 min (**c**), 15 min (**d**), 30 min (**e**). Data are the means ± SE of the evoked $[Ca^{2+}]_{cyt}$ dynamics integrated over 30 min in 6 independent seedlings. Statistical analyses were performed according to Student's *t* test ($P < 0.05$), with different letters indicating significant differences.

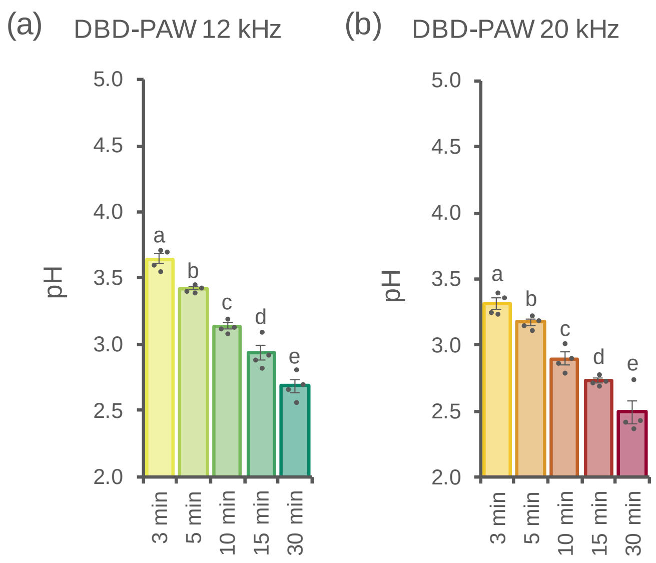

Figure S3. Measurements of DBD-PAW acidity. pH analyses were performed on DBD-PAWs obtained at different discharge frequency: (**a**) 12 kHz (green shades); (**b**) 20 kHz (red shades). DBD-PAWs were collected by progressively increasing the exposure time of deionized H_2O to the DBD-cold plasma (from 3 to 30 min). Data are the means ± SE of 4 independent samples. Statistical analyses were performed according to Student's *t* test ($P < 0.05$), with different letters indicating significant differences.

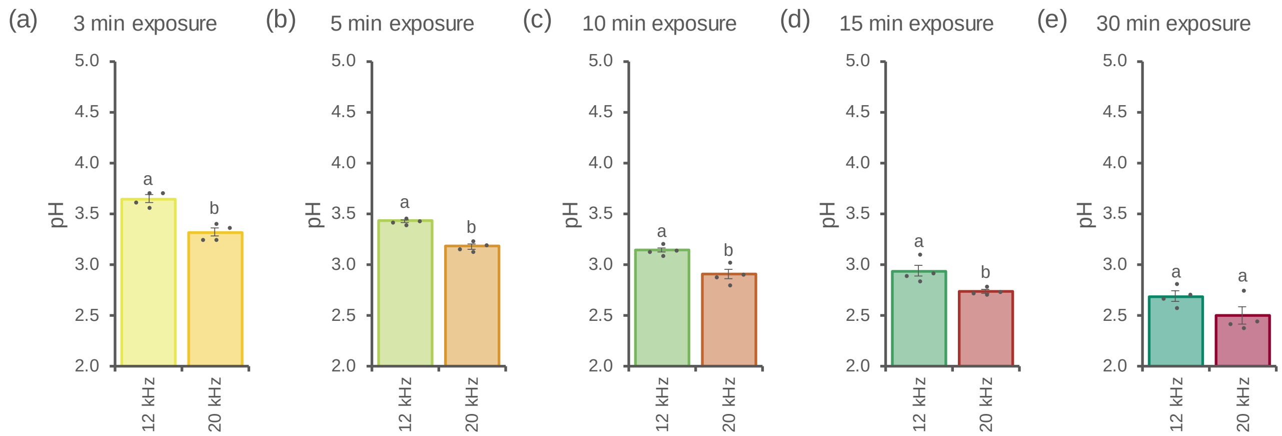

Figure S4. Comparative analysis of DBD-PAWs acidity in relation to two different plasma discharge frequencies used in their generation. DBD plasma discharge frequency was set either at 12 kHz (green shades) or 20 kHz (red shades) and for each setting five different DBD-PAWs were obtained by exposing deionized H_2O to DBD-cold plasma for increasing time intervals: 3 min (**a**), 5 min (**b**), 10 min (**c**), 15 min (**d**), 30 min (**e**). Data are the means ± SE of 4 independent samples. Statistical analyses were performed according to Student's *t* test ($P < 0.05$), with different letters indicating significant differences.

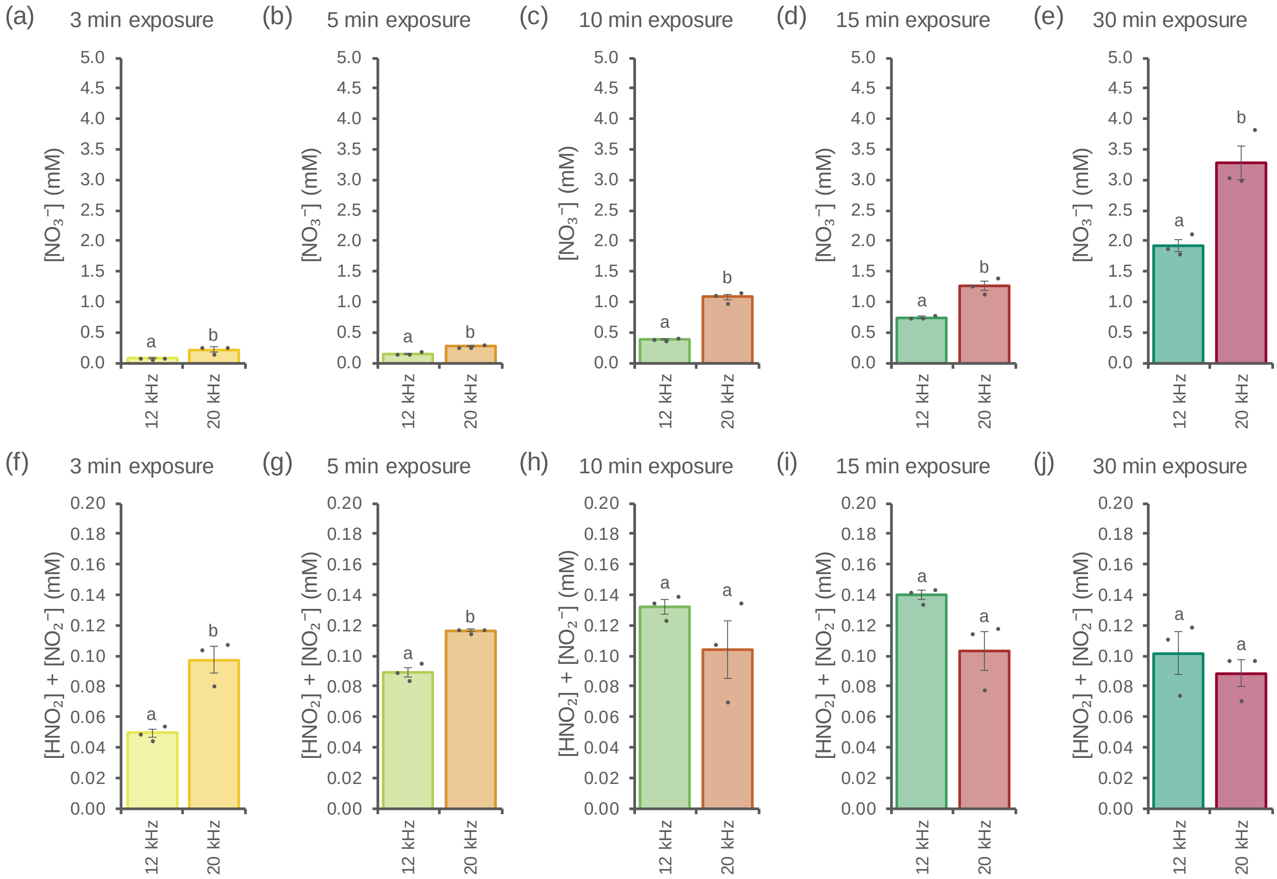

Figure S5. Comparative quantification analyses of the main nitrogen species in DBD-PAWs in relation to the different plasma discharge frequencies used in their generation. Ion chromatography analyses were performed on various DBD-PAWs, which discharge frequency was set either at 12 kHz (green shades) or 20 kHz (red shades). For each setting five different DBD-PAWs were obtained by exposing deionized H_2O to DBD-cold plasma for increasing time intervals: 3 min (**a**,**f**), 5 min (**b**,**g**), 10 min (**c**,**h**), 15 min (**d**,**i**), 30 min (**e**,**j**). Data are the means ± SE of 3 independent samples. Statistical analyses were performed according to Student's *t* test ($P < 0.05$), with different letters indicating significant differences.
